# Hybrid fixed point theorems of graphic contractions with applications

**DOI:** 10.1016/j.heliyon.2024.e31269

**Published:** 2024-05-16

**Authors:** Jamilu Abubakar Jiddah, Mohammed Shehu Shagari, Maha Noorwali, Ahmad Aloqaily, Nabil Mlaiki

**Affiliations:** a Department of Mathematics, School of Physical Sciences, Federal University of Technology, Minna, Nigeria; b Department of Mathematics, Faculty of Physical Sciences Ahmadu Bello University, Zaria, Nigeria; c Department of Mathematics, King Abdulaziz University Jeddah, Saudi Arabia; d Department of Mathematics and Sciences, Prince Sultan University, Riyadh 11586, Saudi Arabia; e School of Computer, Data and Mathematical Sciences, Western Sydney University, Sydney, 2150, Australia

**Keywords:** 46T99, 47G10, 46N40, 54G25, 05C40, Fixed point, Jaggi-type contractive mapping, Hybrid contractive mapping, Graphic contraction, Picard operator, Ulam stability

## Abstract

In this work, a family of hybrid contractions, termed Jaggi-type hybrid (ℵ-*τ*)-contractive mapping is proposed in metric space equipped with a graph and new conditions under which the mapping is a Picard operator are studied. The novel ideas proposed in this manuscript are exemplified to display the validity of the presented results and to show how they differ from the existing ones. Additionally, some corollaries which reduce our proposed notion to some recently announced concepts in the existing findings are indicated and examined. Finally, we study Ulam-type stability for the fixed point equations with hybrid contractions.

## 1 Introduction and preliminaries

The Banach contraction principle (BCP) is a powerful concept in investigating fixed points (Fp) in the framework of metric spaces (MS), (see, e.g. [1], [2], [3]). This principle is particularly useful because it has practical applications in various fields, including applied mathematics, engineering, and even social sciences. Lately, research has focused on the behavior of the *Fp* results for rational and non-rational contractions within the context of metric spaces (see e.g. [4], [7], [8], [13]). Building on the (BCP), Jaggi [18] launched a new and more general concept in 1977.

Definition 1.1 [18]. Presume (μ,υ) to be a MS. Consider a self-mapping Ω of *μ* is termed a Jaggi contractive mapping if we can obtain $\lambda_{1},\lambda_{2}\in [0,1)$ with $\lambda_{1}+\lambda_{2}<1$ such that for all discrete points s,ħ∈μ,

$$\upsilon (\Omega s,\Omega \hbar )\leq \lambda_{1}\frac{\upsilon (s,\Omega s)+\upsilon (\hbar ,\Omega \hbar )}{\upsilon (s,\hbar )}+\lambda_{2}\upsilon (s,\hbar ).$$

As reported by Petruşel and Rus [34], a Picard operator (OP) is a mapping Ω of a MS (μ,υ) that has a unique *Fp* $s^{⁎}$ and $\lim_{j\to \infty }\Omega^{j}s=s^{⁎}$ for all s∈μ. Ω is termed a weakly PO if ${\left\{\Omega^{j}s\right\}}_{j\in N}$ is convergent for every s∈μ and the limit is a *Fp* of Ω.

Jachymski [17] came up with the concept of a graphic contractive mapping in MS. Assume (μ,υ) is a MS and ϒ, the diagonal of the Cartesian product μ×μ. Take a directed graph ℵ in the sense that its vertices set U(ℵ) is given by *μ*, and its edges set, D(ℵ). The loops set, ϒ is a subset of D(ℵ). Assume that ℵ does not contain parallel edges. Then ℵ can be represented as (U(ℵ),D(ℵ)). In addition, ℵ can be seen to be a weighted graph if we assign to each edge the distance between its vertices (see [[22], p. 376]). A graph ${ℵ}^{-1}$ is obtained from ℵ if the directions of edges of ℵ are reversed. In that case,

(1.1) $$D({ℵ}^{-1})=\{(s,\hbar )\in \mu \times \mu |(\hbar ,s)\in D(ℵ)\}.$$

In the same way, when the directions of edges of ℵ are neglected or when the set of edges is symmetric, the undirected graph $\overset{˜}{ℵ}$ is procured. In that case,

(1.2) $$D(\overset{˜}{ℵ})=D(ℵ)\cup D({ℵ}^{-1}).$$

A subgraph of a graph ℵ is a pair $\left(U^{\prime },E^{\prime }\right)$ if $U(ℵ)⊇U^{\prime }$, $D(ℵ)⊇E^{\prime }$ and for each $(s,\hbar )\in E^{\prime }$, $s,\hbar \in U^{\prime }$. Let s,ħ∈U. A sequence ${\left\{s_{i}\right\}}_{i=0}^{J}$ of J+1 vertices satisfying $s_{0}=s$, $s_{J}=\hbar$ and $\left(s_{j-1},s_{j})\in D(ℵ\right)$ for all i=1,2,...,J defines a path in ℵ from *s* to length J∈N. If there is a path for any s,ħ∈U, then ℵ is a connected graph. If $\overset{˜}{ℵ}$ is connected, then ℵ is said to be weakly connected.

In this regard, *Fp* results in MS empowered with graph have been discussed by many investigators (e.g. [5], [10], [11], [12], [14], [16], [17], [19], [21], [31], [36]). Specifically, Bojor [10] launched:

Definition 1.2 [10] Denote by Ψ, the set of all real-valued functions *τ* such that:

(i) *τ* is monotone non-decreasing, i.e., $q_{1}\leq q_{2}$ implies $\tau (q_{1})\leq \tau (q_{2})$;
(ii) $\sum_{j=0}^{\infty }\tau^{j}(q)$ converges for all q>0.

Then *τ* is referred to as (*c*)-comparison function.

Definition 1.3 [10] On a MS (μ,υ) equipped with a graph ℵ, a self-map Ω of *μ* is termed a (ℵ-*τ*)-contractive mapping if:

(i) Ω retains the edges of ℵ, i.e.,
  (s,ħ)∈D(ℵ)⇒(Ωs,Ωħ)∈D(ℵ)∀s,ħ∈μ;
(ii) ∃τ∈Ψ that confirm
  (1.3) υ(Ωs,Ωħ)≤τ(υ(s,ħ))∀(s,ħ)∈D(ℵ).

Definition 1.4 [10] A self-map Ω of *μ* is said to fulfill orbital continuity condition if for all s,ħ∈μ and any sequence ${\left\{k_{j}\right\}}_{j\in N}$, $\Omega^{k_{j}}s⟶\hbar \in \mu$ implies that $\Omega (\Omega^{k_{j}}s)⟶\Omega \hbar$ as j→∞.

Definition 1.5 [10] A self-map Ω of *μ* is said to fulfill orbital ℵ-continuity condition if for all s∈μ and any sequence ${\left\{s_{j}\right\}}_{j\in N}$, $s_{j}⟶s$ with $\left(s_{j},s_{j+1})\in D(ℵ\right)$ imply that $\Omega s_{j}⟶\Omega s$ as j→∞.

Theorem 1.6 *[10]**On a complete MS*(μ,υ)*Endowed with a graph* ℵ*, and a* (ℵ*-τ*)*-contractive mapping* Ω*, if we suppose in addition that:*

(*i*) ℵ *is weakly connected;*
(ii) *every sequence*${\left\{s_{j}\right\}}_{j\in N}$*in μ with*$\upsilon (s_{j},s_{j+1})⟶0$*is such that we can find*$j_{0}\in N$*fulfilling*$\left(s_{j_{k}},s_{j_{m}})\in D(ℵ\right)$*for all*k,m∈N*with*$k,m\geq j_{0}$*;*
${\left(iii\right)}_{a}$ Ω *meets orbital continuity condition or;*
${\left(iii\right)}_{b}$ Ω *meets orbital* ℵ*-continuity condition and there is a subsequence* ${\left\{\Omega^{j_{k}}s_{0}\right\}}_{k\in N}$ *of* ${\left\{\Omega^{j}s_{0}\right\}}_{j\in N}$ *such that* $\left(\Omega^{j_{k}}s_{0},s^{⁎})\in D(ℵ\right)$ *for each* k∈N *and some* $s_{0},s^{⁎}\in \mu$*.*

*Then* Ω *is a PO.*

Karapınar [23] recently propounded a novel type of contractive mapping derived from the definition of Kannan contractive mapping via interpolation. Several academics have utilized this interpolative method to acquire refinement of different types of contractions (see e.g., [9], [24], [25], [26], [30], [32], [35]). In this regard, Karapınar and Fulga [27] presented a new concept of hybrid contractive mapping, which is a combination of some current linear, nonlinear, and interpolative contractions in MS.

Definition 1.7 [27] On a complete MS (μ,υ), a self-map Ω of *μ* is termed a Jaggi-type hybrid (J-type /H) contractive mapping, if we can find τ∈Ψ such that:

(1.4) υ(Ωs,Ωħ)≤τ(J(s,ħ)),

for all distinct s,ħ∈μ, where

$$J(s,\hbar )=\left\{ \begin{array}{c} {\left[\lambda_{1}{\left(\frac{\upsilon (s,\Omega s)\cdot \upsilon (\hbar ,\Omega \hbar )}{\upsilon (s,\hbar )}\right)}^{℘}+\lambda_{2}\upsilon {\left(s,\hbar \right)}^{℘}\right]}^{\frac{1}{℘}},\\ \;\;\;\;\;\;\;for\;℘>0,\;s,\hbar \in \mu ,s\neq \hbar ;\\ \upsilon {\left(s,\Omega s\right)}^{\lambda_{1}}\cdot \upsilon {\left(\hbar ,\Omega \hbar \right)}^{\lambda_{2}},\;\;\;for\;℘=0,\;s,\hbar \in \mu ﹨Fix(\Omega ), \end{array}\right.$$

$\lambda_{1},\lambda_{2}\geq 0$ with $\lambda_{1}+\lambda_{2}=1$ and Fix(Ω)={s∈μ:Ωs=s}.

The chief role of hybrid contractions is that they allow for the presentation of contractive conditions involving a substantial amount of terms, including certain ones with self-composition of the mapping, while also admitting a number of parameters, allowing for extensions in various ways based on the parameters picked. We refer to [6], [15], [20], [27], [29], [33] and the references therein for various improvements on this matter. In accordance with the existing findings, we notice that hybrid *Fp* concepts in MS equipped with graph have not been exhaustively studied. Hence, inspired by the ideas in [10], [13], [17], [27], [28], we initiate a novel view of J-type /H (ℵ-*τ*)-contractive mapping in MS equipped with a graph and study the criteria under which the mapping is a PO. Comparative illustrations are set up to show that our derived results are genuine and different from the existing ones. Moreover, some consequences are noted to indicate that the ideas launched in this work add up and complement some corresponding results.

Below here, we consider *μ* as non-empty. The symbols N, R, and $R_{+}$ Symbolize the sets of natural numbers, real numbers, and non-negative real numbers respectively.

## 2 Main results

In this section, a novel idea of J-type /H (ℵ-*τ*)-contractive mapping in MS characterized by a graph ℵ is presented.

Definition 2.1 On a MS (μ,υ) characterized by a graph ℵ, a self-mapping Ω of *μ* is termed a J-type /H (ℵ-*τ*)-contractive mapping if:

(i) Ω maintains the edges of ℵ;
(ii) ∃τ∈Ψ which verifies
  (2.1) υ(Ωs,Ωħ)≤τ(J(s,ħ))
  for all (s,ħ)∈D(ℵ), where
  $$J(s,\hbar )=\left\{ \begin{array}{c} {\left[\lambda_{1}{\left(\frac{\upsilon (s,\Omega s)\cdot \upsilon (\hbar ,\Omega \hbar )}{\upsilon (s,\hbar )}\right)}^{℘}+\lambda_{2}\upsilon {\left(s,\hbar \right)}^{℘}\right]}^{\frac{1}{℘}},\\ \;\;\;\;\;\;\;\mathrm{for}\;\;\mathrm{some}\;℘>0,\;s\neq \hbar ;\\ \upsilon {\left(s,\Omega s\right)}^{\lambda_{1}}\cdot \upsilon {\left(\hbar ,\Omega \hbar \right)}^{\lambda_{2}},\;\mathrm{for}\;℘=0,\;\{s,\hbar \}⊄Fix(\Omega ), \end{array}\right.$$
  Fix(Ω)={s∈μ:Ωs=s} and $\lambda_{1},\lambda_{2}\geq 0$ with $\lambda_{1}+\lambda_{2}=1$.

Example 2.2 Let μ={s|s≤4,s∈N} along with the metric υ(s,ħ)=|s−ħ| ∀s,ħ∈μ. Consider a self-map Ω on *μ* defined by

$$\Omega s=\left\{ \begin{array}{cc} 2s, & \text{if }s=1;\\ s, & \text{if }s=2;\\ 1, & \text{if }s=3;\\ \frac{s}{2}, & \text{if }s=4. \end{array}\right.$$

**Figure 1:**
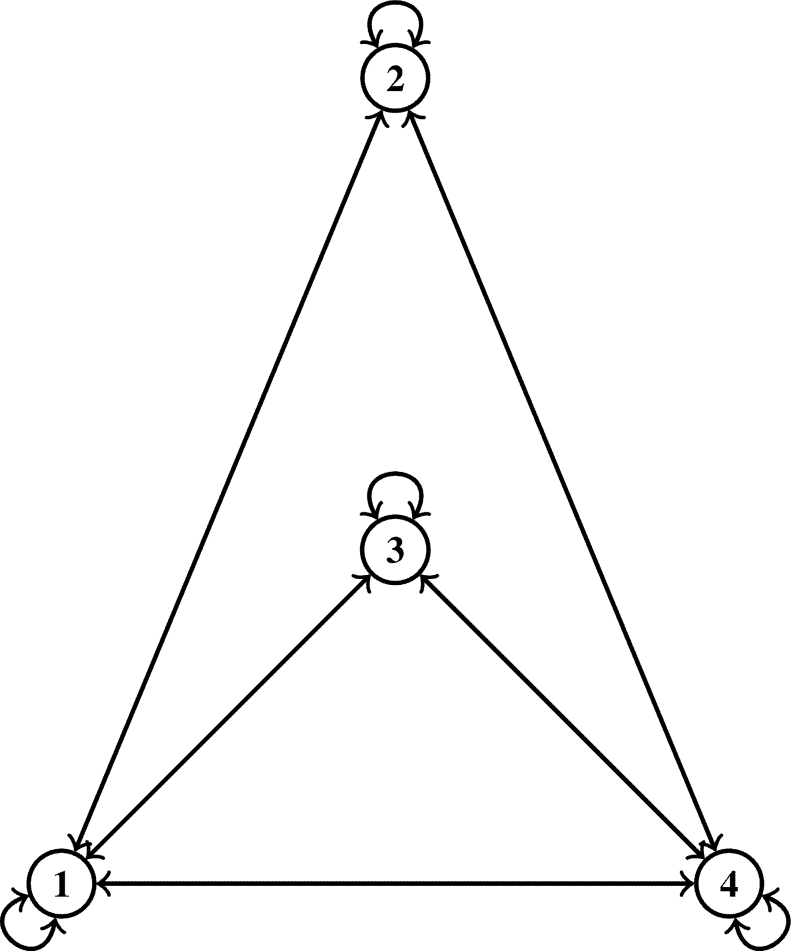
Symmetric graph $\overset{˜}{ℵ}$ given in Example 2.2.

Then Ω is a J-type /H (ℵ-*τ*)-contractive mapping with $\tau (t)=\frac{4t}{5}$, $\lambda_{1}=\frac{2}{5}$ and $\lambda_{2}=\frac{3}{5}$ for ℘=0,5, where $\overset{˜}{ℵ}$ is a symmetric graph such that $U(\overset{˜}{ℵ})=\mu$ and

$$D(\overset{˜}{ℵ})=\{(1,2),(1,3),(1,4),(2,4),(3,4)\}\cup ϒ,$$

but Ω doesn't satisfy the J-type /H contractive mapping as given in [27], given that υ(Ω2,Ω3)=1 while τ(J(2,3))=0 for ℘=0 and for ℘=5, $\tau (J(2,3))\approx \frac{18}{25}$, that is,

$$\tau (J(2,3))=\;\frac{4}{5}\left({\left[\frac{2}{5}{\left(\frac{\upsilon (2,\Omega (2))\cdot \upsilon (3,\Omega (3))}{\upsilon (2,3)}\right)}^{5}+\frac{3}{5}\upsilon {\left(2,3\right)}^{5}\right]}^{\frac{1}{5}}\right)=\;\frac{4}{5}\left({\left[\frac{2}{5}{\left(\frac{\upsilon (2,2)\cdot \upsilon (3,1)}{\upsilon (2,3)}\right)}^{5}+\frac{3}{5}\upsilon {\left(2,3\right)}^{5}\right]}^{\frac{1}{5}}\right)=\;\frac{4}{5}\left({\left[\frac{2}{5}{\left(\frac{0\cdot 2}{1}\right)}^{5}+\frac{3}{5}{\left(1\right)}^{5}\right]}^{\frac{1}{5}}\right)=\;\frac{4}{5}\left({\left(\frac{3}{5}\right)}^{\frac{1}{5}}\right)=0.72230436\approx 0.72=\frac{18}{25}.$$

(Fig. 1) is the symmetric graph given in Example 2.2.

We proceed with the proof of our main result.

Theorem 2.3 *On a complete MS*(μ,υ)*characterized by a graph* ℵ*, and a J-type /H* (ℵ*-τ*)*-contractive mapping* Ω*. Additionally, assume:*

(*i*) ℵ *is weakly connected;*
(ii) *for all sequence*${\left\{s_{j}\right\}}_{j\in N}$*in μ with*$\upsilon (s_{j},s_{j+1})⟶0$*is such that we can identify*$j_{0}\in N$*satisfying*$\left(s_{j_{k}},s_{j_{m}})\in D(ℵ\right)$*for all*k,m∈N*with*$k,m\geq j_{0}$*;*
${\left(iii\right)}_{a}$ Ω *meets orbital continuity condition or;*
${\left(iii\right)}_{b}$ Ω *meets orbital* ℵ*-continuity condition and there is a subsequence* ${\left\{\Omega^{j_{k}}s_{0}\right\}}_{k\in N}$ *of* ${\left\{\Omega^{j}s_{0}\right\}}_{j\in N}$ *such that* $\left(\Omega^{j_{k}}s_{0},s^{⁎})\in D(ℵ\right)$ *for each* k∈N *and some* $s_{0},s^{⁎}\in \mu$*.*

*Then* Ω *is a PO.*

Proof Consider $s_{0}\in \mu$ with $\left(s_{0},\Omega s_{0})\in D(ℵ\right)$ and define a sequence ${\left\{s_{j}\right\}}_{j\in N}$ by $s_{j}=\Omega^{j}s_{0}$. Following a well-proposed inductive approach, we can establish that $\left(\Omega^{j}s_{0},\Omega^{j+1}s_{0})\in D(ℵ\right)$. By (2.1), we have

(2.2) $$\upsilon (\Omega s_{j-1},\Omega s_{j})\leq \tau (J(s_{j-1},s_{j}))$$

Considering Case 1 of (2.1), we have

$$J(s_{j-1},s_{j})=\;{\left[\lambda_{1}{\left(\frac{\upsilon (s_{j-1},\Omega s_{j-1})\cdot \upsilon (s_{j},\Omega s_{j})}{\upsilon (s_{j-1},s_{j})}\right)}^{℘}+\lambda_{2}\upsilon {\left(s_{j-1},s_{j}\right)}^{℘}\right]}^{\frac{1}{℘}}=\;{\left[\lambda_{1}{\left(\frac{\upsilon (s_{j-1},s_{j})\cdot \upsilon (s_{j},s_{j+1})}{\upsilon (s_{j-1},s_{j})}\right)}^{℘}+\lambda_{2}\upsilon {\left(s_{j-1},s_{j}\right)}^{℘}\right]}^{\frac{1}{℘}}=\;{\left[\lambda_{1}\upsilon {\left(s_{j},s_{j+1}\right)}^{℘}+\lambda_{2}\upsilon {\left(s_{j-1},s_{j}\right)}^{℘}\right]}^{\frac{1}{℘}}.$$

Hence, (2.2) becomes

$$\upsilon (\Omega s_{j-1},\Omega s_{j})\leq \;\tau \left({\left[\lambda_{1}\upsilon {\left(s_{j},s_{j+1}\right)}^{℘}+\lambda_{2}\upsilon {\left(s_{j-1},s_{j}\right)}^{℘}\right]}^{\frac{1}{℘}}\right).$$

Now, if $\upsilon (s_{j-1},s_{j})\leq \upsilon (s_{j},s_{j+1})$, then we have

$$\upsilon (\Omega s_{j-1},\Omega s_{j})=\upsilon (s_{j},s_{j+1})\leq \;\tau \left({\left[\lambda_{1}\upsilon {\left(s_{j},s_{j+1}\right)}^{℘}+\lambda_{2}\upsilon {\left(s_{j-1},s_{j}\right)}^{℘}\right]}^{\frac{1}{℘}}\right)\leq \;\tau \left({\left[\lambda_{1}\upsilon {\left(s_{j},s_{j+1}\right)}^{℘}+\lambda_{2}\upsilon {\left(s_{j},s_{j+1}\right)}^{℘}\right]}^{\frac{1}{℘}}\right)\leq \;\tau \left({\left[(\lambda_{1}+\lambda_{2})\upsilon {\left(s_{j},s_{j+1}\right)}^{℘}\right]}^{\frac{1}{℘}}\right)=\;\tau \left(\upsilon (s_{j},s_{j+1})\right)<\;\upsilon (s_{j},s_{j+1}),$$

a contradiction. Therefore, $\upsilon (s_{j},s_{j+1})<\upsilon (s_{j-1},s_{j})$, so that (2.2) becomes

$$\upsilon (s_{j},s_{j+1})\leq \;\tau \left(\upsilon (s_{j-1},s_{j})\right).$$

Continuing inductively, we obtain

$$\upsilon (s_{j},s_{j+1})\leq \;\tau^{j}\left(\upsilon (s_{0},\Omega s_{0})\right)\;\forall \;j\in N.$$

Also by Case 2, we have

$$\upsilon (\Omega s_{j-1},\Omega s_{j})\leq \;\tau \left(\upsilon {\left(s_{j-1},\Omega s_{j-1}\right)}^{\lambda_{1}}\cdot \upsilon {\left(s_{j},\Omega s_{j}\right)}^{\lambda_{2}}\right)<\;\upsilon {\left(s_{j-1},\Omega s_{j-1}\right)}^{\lambda_{1}}\cdot \upsilon {\left(s_{j},\Omega s_{j}\right)}^{\lambda_{2}}.$$

Since $\lambda_{1}+\lambda_{2}=1$, then the above inequality yields

$$\upsilon (s_{j},s_{j+1})<\upsilon (s_{j-1},s_{j})\;\forall \;j\in N.$$

Hence, inequality (2.2) becomes

$$\upsilon (s_{j},s_{j+1})\leq \;\tau \left(\upsilon (s_{j-1},s_{j})\right),$$

and by induction, we obtain

$$\upsilon (s_{j},s_{j+1})\leq \;\tau^{j}\left(\upsilon (s_{0},\Omega s_{0})\right)\;\forall \;j\in N.$$

That is,

$$\upsilon (\Omega^{j}s_{0},\Omega^{j+1}s_{0})\leq \tau^{j}\left(\upsilon (s_{0},\Omega s_{0})\right)$$

for every j∈N. Therefore, we have

$$\lim_{j\to \infty }\upsilon (\Omega^{j}s_{0},\Omega^{j+1}s_{0})=0,$$

furthermore, by (ii), we can identify $j_{0}\in N$ such that

$$(\Omega^{j_{k}}s_{0},\Omega^{j_{m}}s_{0})\in D(ℵ)\;\forall \;k,m\in N\;\mathrm{with}\;k,m\geq j_{0}.$$

Since $\upsilon (\Omega^{j_{k}}s_{0},\Omega^{j_{\left(k+1\right)}}s_{0})⟶0$ for all k∈N, so for any given ϵ>0, we can choose N∈N, $N\geq j_{0}$ so that

$$\upsilon (\Omega^{j_{k}}s_{0},\Omega^{j_{\left(k+1\right)}}s_{0})<\epsilon -\tau (\epsilon )\;\text{for all}\;N\in N.$$

Since $\left(\Omega^{j_{k}}s_{0},\Omega^{j_{\left(k+1\right)}}s_{0})\in D(ℵ\right)$, then for any k≥N, we have

$$\upsilon (\Omega^{j_{k}}s_{0},\Omega^{j_{\left(k+2\right)}}s_{0})\leq \;\upsilon (\Omega^{j_{k}}s_{0},\Omega^{j_{\left(k+1\right)}}s_{0})+\upsilon (\Omega^{j_{\left(k+1\right)}}s_{0},\Omega^{j_{\left(k+2\right)}}s_{0})<\;\epsilon -\tau (\epsilon )+\tau^{j}(\upsilon (\Omega^{j_{k}}s_{0},\Omega^{j_{\left(k+1\right)}}s_{0}))<\epsilon .$$

Similarly, since $\left(\Omega^{j_{k}}s_{0},\Omega^{j_{\left(k+2\right)}}s_{0})\in D(ℵ\right)$, then for every k≥N, we have

$$\upsilon (\Omega^{j_{k}}s_{0},\Omega^{j_{\left(k+3\right)}}s_{0})\leq \;\upsilon (\Omega^{j_{k}}s_{0},\Omega^{j_{\left(k+1\right)}}s_{0})+\upsilon (\Omega^{j_{\left(k+1\right)}}s_{0},\Omega^{j_{\left(k+3\right)}}s_{0})<\;\epsilon -\tau (\epsilon )+\tau^{j}(\upsilon (\Omega^{j_{k}}s_{0},\Omega^{j_{\left(k+2\right)}}s_{0}))<\epsilon .$$

Continuing inductively, we see that

$$\upsilon (\Omega^{j_{k}}s_{0},\Omega^{j_{\left(k+m\right)}}s_{0})\leq \epsilon \;\text{for each}\;k,m\in N,\;k,m\geq N.$$

Therefore, ${\left\{\Omega^{j_{k}}s_{0}\right\}}_{k\in N}$ is a Cauchy sequence in (μ,υ), and so by the completeness of (μ,υ), we have $\Omega^{j_{k}}s_{0}⟶s^{⁎}$ as k→∞. Since $\upsilon (\Omega^{j}s_{0},\Omega^{j+1}s_{0})⟶0$ as j→∞, then we have $\Omega^{j}s_{0}⟶s^{⁎}$ as j→∞.

Now for any arbitrary s∈μ, we see that:

1. if $\left(s,s_{0})\in D(ℵ\right)$, then $\left(\Omega^{j}s,\Omega^{j}s_{0})\in D(ℵ\right)$ for each j∈N. Therefore,
  $$\upsilon (\Omega^{j}s,\Omega^{j}s_{0})\leq \tau^{j}(\upsilon (s,s_{0}))\;\forall j\in N.$$
  Letting j→∞ and using the property of *τ*, we have that $\Omega^{j}s⟶s^{⁎}$.
2. if $\left(s,s_{0})\notin D(ℵ\right)$, as a result of (*i*), we can find a path in $\overset{˜}{ℵ}$, ${\left\{{\overset{˜}{s}}_{i}\right\}}_{i=0}^{N}$ from $s_{0}$ to *s* so that ${\overset{˜}{s}}_{0}=s_{0}$, ${\overset{˜}{s}}_{N}=s$ with $\left({\overset{˜}{s}}_{i-1},{\overset{˜}{s}}_{i})\in D(\overset{˜}{ℵ}\right)$ for all i=1,2,...,N. Then, by simple induction, we end up with
  $$(\Omega^{j}{\overset{˜}{s}}_{i-1},\Omega^{j}{\overset{˜}{s}}_{i})\in D(\overset{˜}{ℵ})\;\text{for}\;i=1,2,...,N\;\text{and}\;\upsilon (\Omega^{j}s_{0},\Omega^{j}s)\leq \sum_{i=1}^{N}\tau^{j}(\upsilon ({\overset{˜}{s}}_{i-1},{\overset{˜}{s}}_{i})),$$
  so that $\upsilon (\Omega^{j}s_{0},\Omega^{j}s)⟶0$, which implies $\Omega^{j}s⟶s^{⁎}$.

Therefore, for every s∈μ, a unique point $s^{⁎}\in \mu$ exists so that

$$\lim_{j\to \infty }\Omega^{j}s=s^{⁎}.$$

To see that $s^{⁎}\in Fix(\Omega )$, if ${\left(iii\right)}_{a}$ holds, it is evident that, $s^{⁎}\in Fix(\Omega )$. Alternatively if ${\left(iii\right)}_{b}$ holds, consequently ${\left\{\Omega^{j_{k}}s_{0}\right\}}_{k\in N}⟶s^{⁎}$ and $\left(\Omega^{j_{k}}s_{0},s^{⁎})\in D(ℵ\right)$, then since Ω satisfies the orbital ℵ-continuity condition, we have $\Omega^{j_{k}+1}s_{0}⟶\Omega s^{⁎}$ as k→∞. Therefore, $\Omega s^{⁎}=s^{⁎}$.

Assuming the existence of ħ∈μ satisfying Ωħ=ħ, therefore, based on the foregoing, we get $\Omega^{j}\hbar ⟶s^{⁎}$, this implies that $\hbar =s^{⁎}$. Hence, Ω is a PO. □

Example 2.4 Let μ={s|s≤6,s∈N} be prepared with the Euclidean metric υ(s,ħ)=|s−ħ| for all s,ħ∈μ. Then (μ,υ) is a complete MS. Consider Ω to be a self-mapping on *μ* defined by

$$\Omega s=\left\{ \begin{array}{cc} \frac{s}{2}, & \text{if }s\in \{2i:i=\bar{1,3}\};\\ 1, & \text{if }s\in \{2i-1:i=\bar{1,3}\} \end{array}\right.$$

for all s∈μ.

Define a symmetric graph $\overset{˜}{ℵ}$ such that $U(\overset{˜}{ℵ})=\mu$ and

$$D(\overset{˜}{ℵ})=\{(1,2),(1,3),(2,3),(2,4),(3,4),(3,6),(4,5),(4,6),(5,6)\}\cup ϒ.$$

Obviously, Ω is edge preserving and ℵ is weakly connected.

Next, we display that Ω is a J-type /H (ℵ-*τ*)-contractive mapping. Given that $\tau (t)=\frac{7t}{8}$ for all t≥0, $\lambda_{1}=\frac{3}{5}$ and $\lambda_{2}=\frac{2}{5}$ for ℘=0,3. The following scenarios are considered:

Case 1: $s,\hbar \in \{2i:i=\bar{1,3}\}$, s=ħ;
Case 2: $s,\hbar \in \{2i:i=\bar{1,3}\}$, s≠ħ;
Case 3: $s,\hbar \in \{2i-1:i=\bar{1,3}\}$, s=ħ;
Case 4: $s,\hbar \in \{2i-1:i=\bar{1,3}\}$, s≠ħ;
Case 5: $s\in \{2i:i=\bar{1,3}\}$ and $\hbar \in \{2i-1:i=\bar{1,3}\}$;
Case 6: $s\in \{2i-1:i=\bar{1,3}\}$ and $\hbar \in \{2i:i=\bar{1,3}\}$.

It will be displayed using the following Table 1 that contractive condition (2.1) is verified for each of the aforementioned instance.

**Table 1:** Verification of contractive inequality (2.1).

Cases	*s*	*ħ*	υ(Ωs,Ωħ)	τ(J(s,ħ)), ℘=0	τ(J(s,ħ)), ℘=3
Case 1	2	2	0	0.875	-
	4	4	0	1.75	-
	6	6	0	2.625	-

Case 2	2	4	1	1.15456	1.36542
	4	2	1	1.32625	1.36542
	4	6	1	2.05813	2.35112
	6	4	1	2.23199	2.35112

Case 3	3	3	0	1.75	-
	5	5	0	3.5	-

Case 4	1	3	0	-	1.28941
	3	1	0	-	1.28941

Case 5	2	1	0	-	0.64470
	2	3	0	1.15456	1.51591
	4	3	1	1.75	2.96222
	4	5	1	2.30913	5.90659
	6	3	2	2.23199	2.18633
	6	5	2	2.94513	8.85718

Case 6	1	2	0	-	0.64470
	3	2	0	1.32625	1.51591
	3	4	1	1.75	2.96222
	3	6	2	2.05813	2.18633
	5	4	1	2.65250	5.90659
	5	6	2	3.11955	8.85718

It can be seen in the above Table 1 that for each of Cases 1−6, υ(Ωs,Ωħ)≤τ(J(s,ħ)) for all $\left(s,\hbar )\in D(\overset{˜}{ℵ}\right)$ as indicated by Columns 4, 5 and 6.

**Figure 2:**
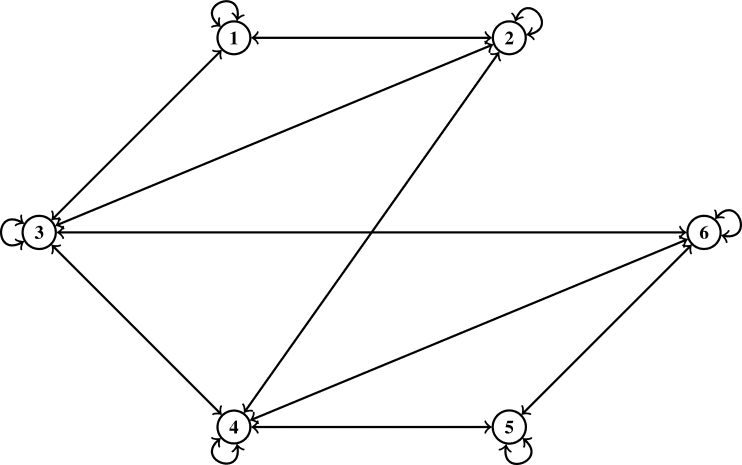
Symmetric graph $\overset{˜}{ℵ}$ given in Example 2.4.

**Figure 3:**
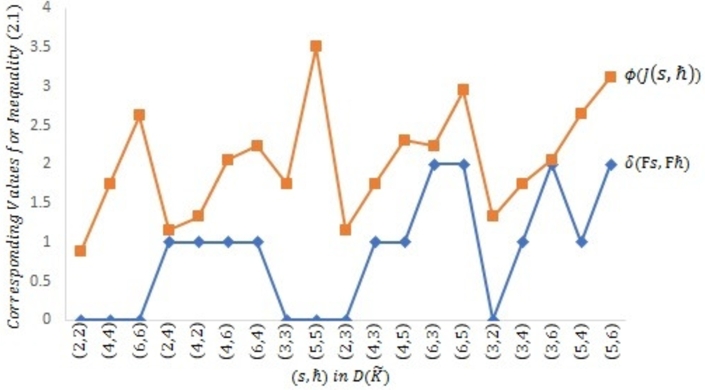
Verification of contractive condition (2.1) for ℘ = 0.

**Figure 4:**
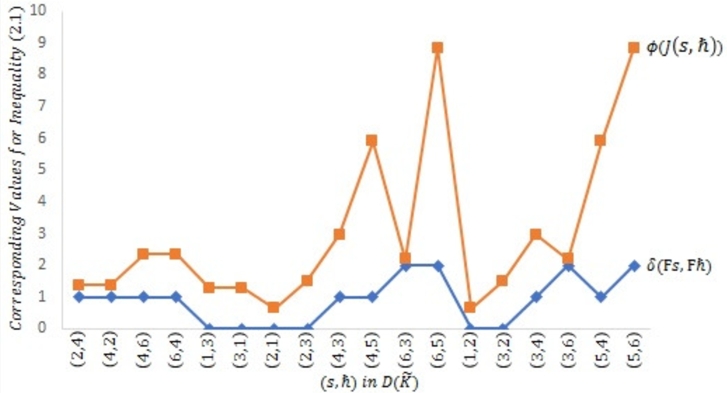
Verification of contractive condition (2.1) for ℘ = 3.

Fig. 2 as depicted below the symmetric graph $\overset{˜}{ℵ}$ for Example 2.4, while Figure 3, Figure 4 further attest that contractive condition (2.1) holds true for Example 2.4.

For ℘=0 and ℘=3 respectively, Figure 3, Figure 4 have shown that υ(Ωs,Ωħ)≤τ(J(s,ħ)) for all $\left(s,\hbar )\in D(\overset{˜}{ℵ}\right)$ as given in Example 2.4.

Therefore, all the hypotheses regarding Theorem 2.3 have been confirmed, there is a unique point s=1 such that Ωs=s, and $\lim_{j\to \infty }\Omega^{j}s=1$ for all s∈μ. In light of this, Ω is a PO.

Subsequently, we display that Theorem 3.2 obtained in [17] can be formulated from our main results. According to [17], consider Ω to be a self-mapping on *μ*. A collection of all points s∈μ fulfilling (s,Ωs)∈D(ℵ) is denoted by $\mu_{\Omega }$, that is,

$$\mu_{\Omega }=\{s\in \mu :(s,\Omega s)\in D(ℵ)\}.$$

Definition 2.5 [17]. On a MS (μ,υ) Incorporated with a graph ℵ, a self-map Ω of *μ* is termed a Banach ℵ-contractive mapping (or simply an ℵ-contractive mapping) if:

(i) Ω conserves the edges of ℵ;
(ii) ∃μ∈(0,1)∀s,ħ∈μsuch that(s,ħ)∈D(ℵ)⇒υ(Ωs,Ωħ)≤μυ(s,ħ).

Corollary 2.6 *(see [**[17]**, Theorem* 3.2*]). On a complete MS* (μ,υ) *characterized by a graph* ℵ*, and a* ℵ*-contractive mapping* Ω*. Consider further that:*

(i) $\mu_{\Omega }\neq \emptyset$*and* ℵ *is weakly connected;*
(ii) *for each sequence*${\left\{s_{j}\right\}}_{j\in N}$*in μ, if*$s_{j}⟶s$*and*$\left(s_{j},s_{j+1})\in D(ℵ\right)$*for all*j∈N*, then we can find subsequence*${\left\{s_{k_{n}}\right\}}_{k\in N}$*with*$\left(s_{k_{n}},s)\in D(ℵ\right)$*for each*k∈N*.*

*Then* Ω *is a PO.*

Proof Take into account Definition 2.1 and given that τ(t)=μt for all t≥0, μ∈(0,1), ℘ > 0, $\lambda_{1}=0$ and $\lambda_{2}=1$. Then J-type /H (ℵ-*τ*)-contractive mapping becomes ℵ-contractive mapping due to Jachymski [17]. Hence, the proof is immediate from Theorem 3.2 of Jachymski [17]. □

Hereafter Corollary 2.7 displays that Theorem 1.6 due to [10] is derivable from our main results.

Corollary 2.7 *On a complete MS*(μ,υ)*characterized by a graph* ℵ*, let* Ω:μ⟶μ *be a* (ℵ*-τ*)*-contractive mapping which verifies all the hypotheses of* Theorem 1.6*. Then* Ω *is a PO.*

Proof Assume in Definition 2.1 that ℘ > 0, $\lambda_{1}=0$ and $\lambda_{2}=1$. Then J-type /H (ℵ-*τ*)-contractive mapping reduces to (ℵ-*τ*)-contractive mapping given by Bojor [10] (see Definition 1.3). Therefore, the proof follows using a similar line of approach. □

Example 2.8 Let μ={s|s≤7,s∈N} be equipped with the Euclidean metric υ(s,ħ)=|s−ħ| for every s,ħ∈μ. Then (μ,υ) is a complete MS. Consider Ω to be a self-mapping on *μ* given by

$$\Omega s=\left\{ \begin{array}{cc} 1, & \text{if }\;1\leq s\leq 2;\\ s-2, & \text{if }\;3\leq s\leq 5;\\ s-5, & \text{if }\;6\leq s\leq 7 \end{array}\right.$$

for all s∈μ.

Define a symmetric graph $\overset{˜}{ℵ}$ such that $V(\overset{˜}{ℵ})=\mu$ and

$$E(\overset{˜}{ℵ})=\{(1,s_{n}),(2,s_{n})\;|\;s_{n}\in \mu ,n\in N\}.$$

Then Ω is edge preserving and orbitally continuous. Also, ℵ is weakly connected.

Now, notice that if $\tau (t)=\frac{3t}{4}$, then

υ(Ω1,Ω1)=υ(Ω1,Ω2)=υ(Ω1,Ω3)=υ(Ω1,Ω6)=0,
$\upsilon (\Omega 1,\Omega 4)=1<\frac{9}{4}=\tau (\upsilon (1,4))$,
υ(Ω1,Ω5)=2<3=τ(υ(1,5)),
$\upsilon (\Omega 1,\Omega 7)=1<\frac{9}{2}=\tau (\upsilon (1,7))$.

Similarly

υ(Ω2,Ω1)=υ(Ω2,Ω2)=υ(Ω2,Ω3)=υ(Ω2,Ω6)=0,
$\upsilon (\Omega 2,\Omega 4)=1<\frac{3}{2}=\tau (\upsilon (2,4))$,
$\upsilon (\Omega 2,\Omega 5)=2<\frac{9}{4}=\tau (\upsilon (2,5))$,
$\upsilon (\Omega 2,\Omega 7)=1<\frac{15}{4}=\tau (\upsilon (2,7))$.

Therefore, Ω is a (ℵ-*τ*)-contractive mapping which satisfies all the assumptions of Theorem 1.6, there is a unique point s=1 such that Ωs=s, and $\lim_{n\to \infty }\Omega^{n}s=1$ for every s∈μ. Thus, Ω is a PO.

**Figure 5:**
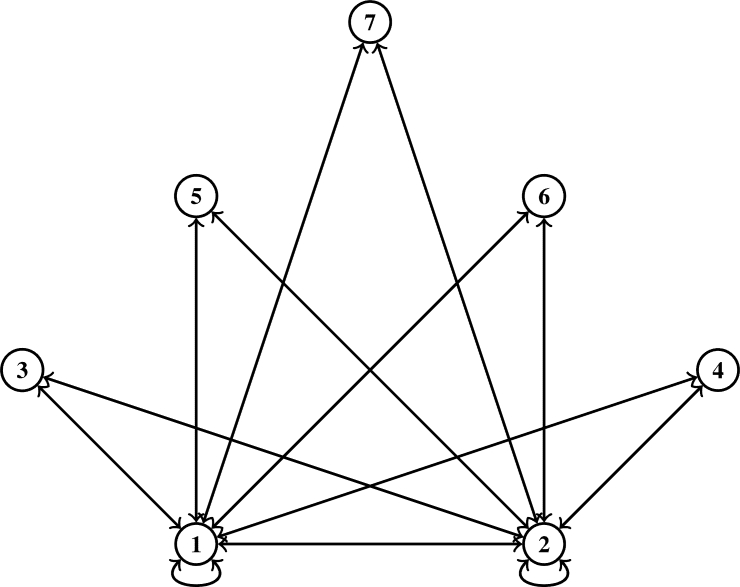
Symmetric graph $\overset{˜}{ℵ}$ given in Example 2.8.

**Figure 6:**
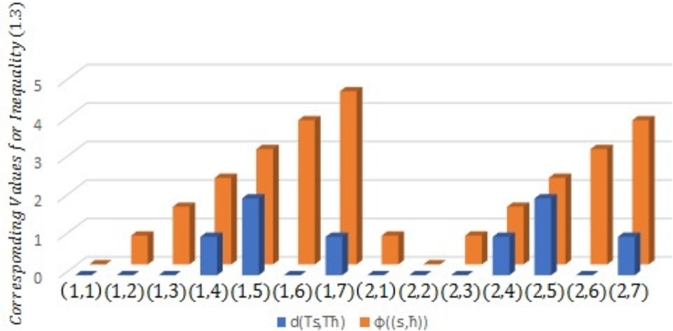
Demonstration of contractive condition (1.3).

Fig. 5 as depicted below the symmetric graph $\overset{˜}{ℵ}$ for Example 2.8, while Fig. 6 visualizes contractive condition of Theorem 1.6.

Fig. 6 has shown that ϑ(Ωs,Ωħ)≤τ(ϑ(s,ħ)) for all $\left(s,\hbar )\in E(\overset{˜}{ℵ}\right)$ as given in 2.8.

## 3 Ulam-type stability

Ulam launched a stability idea, which is a data dependence result. Hyers and other scholars developed this notion further (see [13], [28]). In the context of the *Fp* problem in MS, the general Ulam-type stability was examined by Karapınar and Fulga [28]. In the framework of a MS with a graph, we view the generic Ulam-type stability as a *Fp* problem.

Suppose that Ω:μ⟶μ is a mapping on a MS (μ,υ) characterized by a graph ℵ. Then the *Fp* problem

(3.1) Ωs=s

is of the general Ulam-type stability if and only if we can find an increasing function $\tau :R_{+}⟶R_{+}$, continuous at 0, τ(0)=0 in the sense that for any ϵ>0 and for each $\hbar^{\prime }\in \mu$ verifying the inequality

(3.2) $$\upsilon (\hbar^{\prime },\Omega \hbar^{\prime })\leq \epsilon ,$$

we can find a solution $s^{⁎}\in \mu$ of (3.1) verifying

(3.3) $$\upsilon (s^{⁎},\hbar^{\prime })\leq \tau (\epsilon ).$$

For any C>0, we take τ(t)=Ct for every t≥0. Then the *Fp* of (3.1) is Ulam-type stable.

On a MS (μ,υ) endowed with a graph ℵ, the *Fp* problem (3.1) is termed well-posed if the conditions below are verified:

(i) Ω has a unique *Fp* $s^{⁎}\in \mu$;
(ii) $\upsilon (s_{j},s^{⁎})=0$ for each sequence ${\left\{s_{j}\right\}}_{j\in N}$ in *μ* such that $\upsilon (s_{j},\Omega s_{j})⟶0$ as j⟶∞.

Theorem 3.1 *On a complete MS*(μ,υ)*characterized by a graph* ℵ*, if in addition to the assumptions of* Theorem 2.3*, in the case of* ℘ > 0*, we have* $\left(s^{⁎},\hbar^{\prime })\in D(ℵ\right)$ *for any* $\hbar^{\prime }\in \mu$*,* $\left(s^{⁎},s_{j})\in D(ℵ\right)$ *for each sequence* ${\left\{s_{j}\right\}}_{j\in N}$ *in μ,* $s^{⁎}\in Fix(\Omega )$ *and* $\lambda_{2}\in [0,1)$*, then the conditions below hold:*

(i) *the Fp equation*(3.1)*is Ulam-Hyers stable;*
(ii) *the Fp equation*(3.1)*is well-posed for any*${\left\{s_{j}\right\}}_{j\in N}$*in μ such that*$\lim_{j\to \infty }\upsilon (s_{j},\Omega s_{j})=0$*and*$Fix(\Omega )=\{s^{⁎}\}$*.*

Proof

(i) We displayed in Theorem 2.3, the existence of a unique $s^{⁎}\in \mu$ such that $\Omega s^{⁎}=s^{⁎}$. For a given ϵ>0, let $\hbar^{\prime }\in \mu$ such that
  $$\upsilon (\hbar^{\prime },\Omega \hbar^{\prime })\leq \epsilon .$$
  Then obviously, $s^{⁎}$ satisfies (3.2). Since $\left(s^{⁎},\hbar^{\prime })\in D(ℵ\right)$, then $\left(\Omega s^{⁎},\Omega \hbar^{\prime })\in D(ℵ\right)$. Hence, by the weak connectivity of ℵ and triangle inequality, we have
  $$\upsilon (s^{⁎},\hbar^{\prime })\leq \;\upsilon (s^{⁎},\Omega \hbar^{\prime })+\upsilon (\Omega \hbar^{\prime },\hbar^{\prime })=\;\upsilon (\Omega s^{⁎},\Omega \hbar^{\prime })+\upsilon (\hbar^{\prime },\Omega \hbar^{\prime })\leq \;\tau (J(s^{⁎},\hbar^{\prime }))+\upsilon (\hbar^{\prime },\Omega \hbar^{\prime })<\;J(s^{⁎},\hbar^{\prime })+\upsilon (\hbar^{\prime },\Omega \hbar^{\prime })=\;{\left[\lambda_{1}{\left(\frac{\upsilon (s^{⁎},\Omega s^{⁎})\cdot \upsilon (\hbar^{\prime },\Omega \hbar^{\prime })}{\upsilon (s^{⁎},\hbar^{\prime })}\right)}^{℘}+\lambda_{2}\upsilon {\left(s^{⁎},\hbar^{\prime }\right)}^{℘}\right]}^{\frac{1}{℘}}+\upsilon (\hbar^{\prime },\Omega \hbar^{\prime })=\;{\left[\lambda_{1}{\left(\frac{\upsilon (s^{⁎},s^{⁎})\cdot \upsilon (\hbar^{\prime },\Omega \hbar^{\prime })}{\upsilon (s^{⁎},\hbar^{\prime })}\right)}^{℘}+\lambda_{2}\upsilon {\left(s^{⁎},\hbar^{\prime }\right)}^{℘}\right]}^{\frac{1}{℘}}+\upsilon (\hbar^{\prime },\Omega \hbar^{\prime })=\;\lambda_{2}^{\frac{1}{℘}}\upsilon (s^{⁎},\hbar^{\prime })+\upsilon (\hbar^{\prime },\Omega \hbar^{\prime }),$$
  from which we obtain
  $$\left(1-\lambda_{2}^{\frac{1}{℘}}\right)\upsilon (s^{⁎},\hbar^{\prime })<\upsilon (\hbar^{\prime },\Omega \hbar^{\prime })$$
  implying that
  $$\upsilon (s^{⁎},\hbar^{\prime })<\left(\frac{1}{1-\lambda_{2}^{\frac{1}{℘}}}\right)\upsilon (\hbar^{\prime },\Omega \hbar^{\prime })\leq C\epsilon ,$$
  where $C=\frac{1}{1-\lambda_{2}^{\frac{1}{℘}}}$ for any ℘ > 0 and $\lambda_{2}\in [0,1)$.
(ii) Considering the additional criteria and because $Fix(\Omega )=\{s^{⁎}\}$, we have
  $$\upsilon (s^{⁎},s_{j})\leq \;\upsilon (s^{⁎},\Omega s_{j})+\upsilon (\Omega s_{j},s_{j})=\;\upsilon (\Omega s^{⁎},\Omega s_{j})+\upsilon (s_{j},\Omega s_{j})\leq \;\tau (J(s^{⁎},s_{j}))+\upsilon (s_{j},\Omega s_{j})<\;J(s^{⁎},s_{j})+\upsilon (s_{j},\Omega s_{j})=\;{\left[\lambda_{1}{\left(\frac{\upsilon (s^{⁎},\Omega s^{⁎})\cdot \upsilon (s_{j},\Omega s_{j})}{\upsilon (s^{⁎},s_{j})}\right)}^{℘}+\lambda_{2}\upsilon {\left(s^{⁎},s_{j}\right)}^{℘}\right]}^{\frac{1}{℘}}+\upsilon (s_{j},\Omega s_{j})=\;{\left[\lambda_{1}{\left(\frac{\upsilon (s^{⁎},s^{⁎})\cdot \upsilon (s_{j},\Omega s_{j})}{\upsilon (s^{⁎},s_{j})}\right)}^{℘}+\lambda_{2}\upsilon {\left(s^{⁎},s_{j}\right)}^{℘}\right]}^{\frac{1}{℘}}+\upsilon (s_{j},\Omega s_{j})=\;\lambda_{2}^{\frac{1}{℘}}\upsilon (s^{⁎},s_{j})+\upsilon (s_{j},\Omega s_{j}),$$
  from which we obtain
  $$\left(1-\lambda_{2}^{\frac{1}{℘}}\right)\upsilon (s^{⁎},s_{j})<\upsilon (s_{j},\Omega s_{j})$$
  implying that
  $$\upsilon (s^{⁎},s_{j})<\left(\frac{1}{1-\lambda_{2}^{\frac{1}{℘}}}\right)\upsilon (s_{j},\Omega s_{j}).$$
  Letting j→∞ and keeping in mind that $\lim_{j\to \infty }\upsilon (s_{j},\Omega s_{j})=0$, we obtain
  $$\lim_{j\to \infty }\upsilon (s^{⁎},s_{j})\leq \lim_{j\to \infty }\upsilon (s_{j},\Omega s_{j})=0.$$
  That is, the *Fp* equation (3.1) is well-posed. □

## 4 Conclusion

The idea of J-type /H (ℵ-*τ*)-contractive mapping in MS characterized with a graph is launched in this paper (Definition 2.1). Sufficient criteria under which such a mapping is a PO are investigated (Theorem 2.3). Contrasting examples with graphical illustrations are built to validate the assumptions of our theorems and to display that the new notions can be generalized (Example 2.2, Example 2.4). Corollary 2.6, Corollary 2.7 are provided to display that the approach described herein is a generalization and improvement on several famous results in the literature. In addition, for the contractive mappings presented here, their well-posedness and Ulam-type stability were investigated . The results in this paper are influenced by and compared to [10], [13], [17], [27], [28].

## Funding

This research work is funded by 10.13039/501100012639Prince Sultan University.

## CRediT authorship contribution statement

**Jamilu Abubakar Jiddah:** Writing – original draft, Methodology, Formal analysis. **Mohammed Shehu Shagari:** Writing – review & editing, Investigation, Formal analysis, Conceptualization. **Maha Noorwali:** Methodology, Formal analysis, Conceptualization. **Ahmad Aloqaily:** Writing – review & editing, Investigation, Formal analysis. **Nabil Mlaiki:** Writing – review & editing, Methodology, Formal analysis.

## Declaration of Competing Interest

The authors declare the following financial interests/personal relationships which may be considered as potential competing interests: Nabil Mlaiki reports financial support was provided by 10.13039/501100012639Prince Sultan University, Riyadh 11586, Saudi Arabia. If there are other authors, they declare that they have no known competing financial interests or personal relationships that could have appeared to influence the work reported in this paper.

## Data Availability

No Data were used to support this study.
